# Prepectoral no mesh versus mesh immediate implant-based reconstruction after mastectomy (Restore-B): a multicentre single-blinded randomised controlled feasibility study protocol

**DOI:** 10.1136/bmjopen-2025-103278

**Published:** 2025-10-17

**Authors:** Rachel Rolph, Sue Ziebland, Jonathan Alistair Cook, Cynthia Iglesias, Joanna Wakefield-Scurr, Charles Malyon, Jessica Scaife, Amy Taylor, Aimee Hennessy, Sarah Markham, Marcelle Bernstein, Michael Douek, Restore-B Feasibility Collaborative Group, Pankaj Roy

**Affiliations:** 1 Nuffield Department of Surgical Sciences, University of Oxford, Oxford, UK; 2 Medical Sociology and Health Experiences Research Group, Nuffield Department of Primary Care Health Sciences, University of Oxford, Oxford, UK; 3 Centre for Statistics in Medicine, Nuffield Department of Orthopaedic, Rheumatology and Musculoskeletal Sciences, University of Oxford, Oxford, UK; 4 Health Services and Policy Research, Department of Health Sciences, University of York, York, UK; 5 School of Sport, Health and Exercise Science, Spinnaker Building, University of Portsmouth, Portsmouth, UK

**Keywords:** Patients, Breast surgery, Randomized Controlled Trial

## Abstract

**Introduction:**

Breast cancer is common and women requiring mastectomy will be offered a breast reconstruction if they are surgically suitable candidate. Breast reconstruction can be performed at the same time as the mastectomy (immediate) or delayed to a second operation after cancer treatments. The reconstruction can either use the patients’ own tissue to make the breast (autologous) or use a prosthesis to make the breast in the form of a fixed or expandable volume implant (implant-based breast reconstruction, IBBR). Immediate breast reconstruction on top of the chest wall muscles (prepectoral) is performed worldwide. This operation involves the use of a synthetic or biological mesh placed around the implant under the skin. Increasingly, surgeons are performing this technique without the use of mesh. Both techniques, with and without mesh, have not been compared in a head-to-head randomised controlled trial (RCT); therefore, surgeons and patients do not have high quality data to guide their decision making in this area.

**Methods and analysis:**

UK-based pragmatic multicentre randomised controlled feasibility trial. The primary aim is to determine the feasibility of a definitive RCT comparing the clinical and cost-effectiveness of no-mesh versus mesh-assisted prepectoral breast reconstruction. Secondary objectives will explore patient understanding of mesh and willingness to be randomised within an RCT; determine if it is possible to collect data to inform a future economic analysis on the use of mesh in breast reconstruction and determine the feasibility of measuring breast biomechanics pre-surgery and post breast reconstruction surgery. Total number of patients to be included: 40 (20 per arm).

**Ethics and dissemination:**

This study will be conducted in compliance with the Declaration of Helsinki. Ethical approval has been obtained. Ethics Ref: 23/SC/0302; IRAS Project ID: 301 423. The results of this study will be published in a peer-reviewed medical journal, independent of the results, following the Consolidated Standards of Reporting Trials standards for RCTs.

**Trial registration numbers:**

NCT06112977; ISRCTN17470747.

**STRENGTHS AND LIMITATIONS OF THIS STUDY**

- This is a pragmatic well-designed feasibility randomised controlled trial (RCT) which aims to determine if a future RCT is possible to run to determine the role of mesh in breast reconstruction.
- A mixed-methods qualitative and quantitative approach with patient public involvement input throughout the trial processes has been designed to understand all stakeholders’ views. Importantly, participant views on the acceptability of blinding and surgeon views on randomisation will be collected as these may be key barriers to future trial recruitment.
- The data from a feasibility RCT may support the safety and acceptability of the intervention; however, no statistically significant differences can be determined between the two groups of data.
- The study is limited to short-term surgical outcomes, and longer-term surgical and oncological outcome measures will be collected pending further ethical approval and funding.

## Introduction

### Background and rationale

 Breast cancer affects one in eight women.^1^ It impacts the lives of 55 000 women per year in the UK and over 2 million worldwide; 80% will require surgery. Of these, approximately 40% require a therapeutic mastectomy to treat their cancer and most will be offered breast reconstruction.^2^ Patients with genetic mutations predisposing to breast cancer may choose prophylactic bilateral mastectomy and reconstruction also.^3^ Women choosing to reconstruct their breast at the same operation as their mastectomy (immediate reconstruction) can choose to either use their own tissue (autologous) or a prosthesis (implant or expandable implant) to make the breast shape.

The modern technique for immediate implant-based breast reconstruction (IBBR) involves an implant wrapped in mesh to form the breast following the mastectomy. The implant is placed in front of the pectoralis chest wall muscle (prepectoral). Prepectoral reconstruction was developed in 2015 to avoid the morbidity of raising the pectoralis muscle to cover the implant which resulted in upper body weakness and animation deformity associated with the older technique (submuscular).^4^

Mesh was introduced as a substitute to using the pectoralis muscle to cover the implant in the reconstruction. Uncertainty exists regarding the benefits of mesh; however, it is used in the majority of prepectoral reconstructions in the UK.^5^ Reported benefits from mesh are improved cosmesis and providing support and cover to the implant on the chest wall. There is an increasing public awareness of mesh in surgical operations and complications with mesh use.^6^ Surgeons can safely perform the procedure without the use of mesh, and this technique has become more popular in the past few years.^7^

National data indicate that 36% of women will experience complications requiring unplanned interventions following implant-based reconstruction.^8^ Meta-analyses on published data report mesh may increase complication rates in breast reconstruction; however, data in this field are limited by high levels of heterogeneity and retrospective case series.^9^ Cancer patients are on tight treatment pathways; complications from surgery can delay adjuvant treatments, for example, chemotherapy and radiotherapy, potentially adversely impacting long-term oncological outcomes and patient well-being.^10^

There is a need for high-quality evidence in this field at an international level to inform clinical and health policy decisions about the use of mesh in breast reconstruction and facilitate shared surgeon-patient decision making in the breast clinic.

## Objectives

The main purpose of this trial is to determine the feasibility of conducting a single-blinded randomised controlled trial (RCT) comparing no-mesh to mesh-assisted prepectoral IBBR. Feasibility data on the trial will be collected throughout the trial and assessed on completion of the recruitment period. Specifically, the proportion of patients eligible, proportion of patients consented and randomised, proportion of patients lost to follow-up or withdrawn from the study will be quantified and used to inform the assessment of feasibility of a corresponding definitive (‘main’) trial. Patient and clinical equipoise to the two intervention arms and willingness to take part in the trial will also be assessed through a qualitative component involving interviews. The trial will collect data on clinical and patient outcomes to inform a future cost-effectiveness decision model structure for IBBR. Completeness of data per participant at 3 months postoperatively and clinical outcome measures will be collected to inform a future RCT sample size calculation. Traffic light criteria will be applied at the end of the feasibility trial to determine progression to the definitive RCT.

Two optional substudies will be offered to patients:

A qualitative interview with a trial researcher to discuss their views on mesh in IBBR, trial design and barriers to research participation. This interview is open to both women who would like to participate in the trial and those who decide not to take part.

Participants who consented to the main trial will be offered the opportunity to take part in a substudy to determine the feasibility of assessing breast biomechanics pre-IBBR and post-IBBR.

## Methods and analysis

### Patient and public involvement

Patient advocates are members of the trial management committee and trial steering committee and have been involved in all aspects of trial design and conduct. Two members are coauthors on this publication. The design of this trial places patient views at the centre of the study through qualitative interviewing and inclusion of patient ranking of outcome measures within the trial to understand what issues matter most to patients undergoing this breast reconstruction surgery. The results of this study will be broadly broadcast to patients involved in the study via the trial newsletter and general public via publication of results in a peer-reviewed scientific journal, conference presentation and social media.

### Trial design and setting

**Figure 1:**
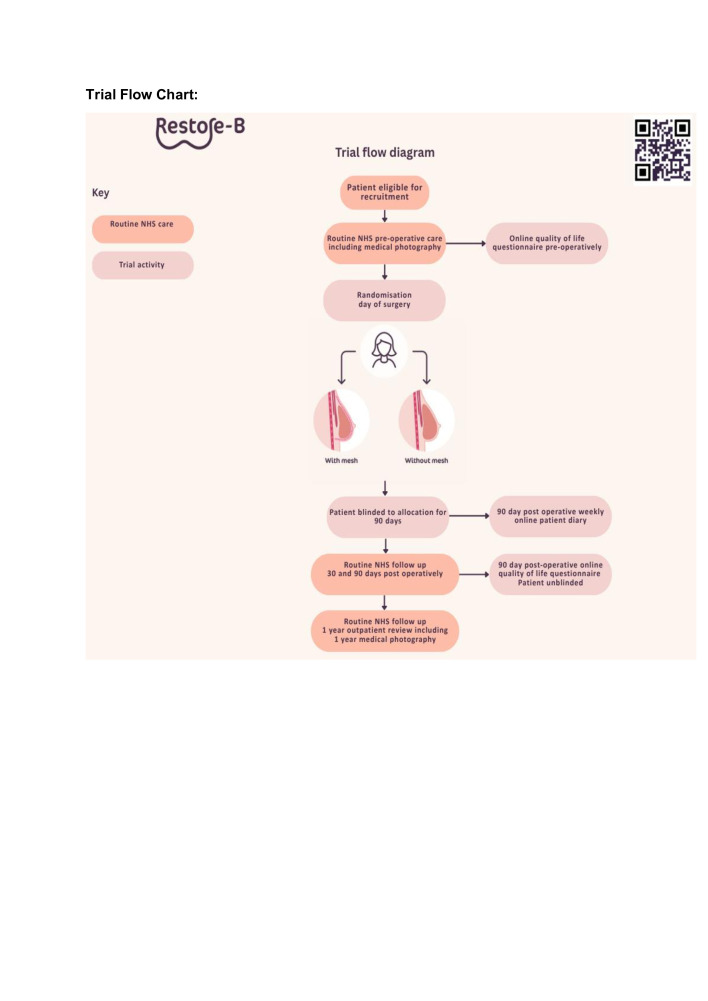
Trial flow chart.

This is a prospective multicentre parallel 2-armed participant-blinded, randomised controlled feasibility trial will compare two surgical techniques for prepectoral IBBR: no-mesh versus mesh-assisted breast reconstruction. The two interventions will be allocated in a 1:1 random allocation at the time of surgery. We will recruit a total of 40 patients from breast reconstruction surgery centres across the UK. The prepectoral breast reconstruction will be performed according to the surgeon’s usual standard of care; the only difference between the two arms will be the placement of mesh or not in the breast reconstruction. Participants will be blinded to their allocation until 90 days postoperatively and will be unblinded after completion of their 90 day postoperative quality of life (QoL questionnaire (figure 1 shows the trial design). The study duration is planned for 12 months with 90 days follow-up for each participant.

### Eligibility criteria

Women with a diagnosis of breast cancer or a gene mutation predisposing to breast cancer undergoing immediate pre-pectoral IBBR will be eligible to participate in the trial. Women must be over 18 years of age, willing and able to give informed consent and able to comply with all trial requirements. Patients are unable to enter the trial if the participant becomes pregnant or is planning pregnancy or breastfeeding; refuses to enter the trial or is undergoing delayed breast reconstruction post simple mastectomy. Both unilateral and bilateral breast reconstructions are included in the trial. Randomisation will take place at the patient level; therefore, a bilateral case will have the same allocation across both reconstructions. Each patient will be informed that participation is voluntary and withdrawal at any point within the trial is supported and would not affect ongoing medical care.

### Intervention and comparator

#### Main trial

The local principal investigator will confirm a patient’s eligibility to participate in the trial having identified patients from their multidisciplinary team meetings and oncoplastic clinics. The trial will be offered to the patient and a written patient information sheet will be given prior to consent. After written informed consent has been obtained by the local principal investigator, patient baseline data will be collected using case report forms (CRFs) accessed via the online database RedCap. CRFs will include collection of routine clinical data including patient and tumour characteristics, comorbidity and cancer treatments and progress through the treatment pathway. This information will be collected as required to describe the cohort and assess the representativeness of those recruited within the study to the general breast cancer and risk-reduction population.

Routine baseline medical photographs and a preoperative patient QoL questionnaire will be sent for completion. This includes the BREAST-Q and EQ5D QoL questionnaires and patient ranking of clinical outcome measures from least important to most important to them which are emailed to participants. QoL questionnaire data will form part of the economic modelling for the two interventions. Preoperative and postoperative photographs will be anonymised and assessed via a blinded patient and professional panel for breast aesthetics between the two groups who will view the images pre- and post-operatively and mark them out of 5 on a Likert scale for cosmesis (RR3).

### Outcomes

The primary endpoint of this study is the feasibility criteria to progress to a future main RCT comparing no mesh to mesh assisted prepectoral IBBR. The main limiting factor for surgical trials is recruitment. A progression dashboard with red/amber/green thresholds is proposed to evaluate the success of the feasibility trial. Achieving all green targets means proceeding to full proof-of-concept is feasible, whereas achieving all red targets would almost certainly indicate that a full proof-of-concept is not feasible. A mixture of green/amber/red thresholds will result in restrategising: recruitment and/or retention strategies will be reconsidered. The primary outcome to be measured for a future RCT will be determined through patient and surgeon ranking of clinical outcome measures and qualitative interviewing of patients to understand what matters the most to patients and surgeons in IBBR.

### Data collection methods

Intraoperative data will be collected including details on the mastectomy technique and volume, the type and volume of prosthesis (±mesh) used in the operation, mastectomy weight, use of sutures, drains and antibiotics.

Preoperative, perioperative and postoperative care will be standard NHS care as per local practice including standard surgical outpatient follow-up at 14 days, 30 days and 90 days. Clinical outcome data will be collected at the same time points as NHS care. Clinical data collected includes clinical history, clinical postoperative complications and patient access to healthcare outside of the tertiary hospital setting via patient weekly online diaries.

**Figure 2:**
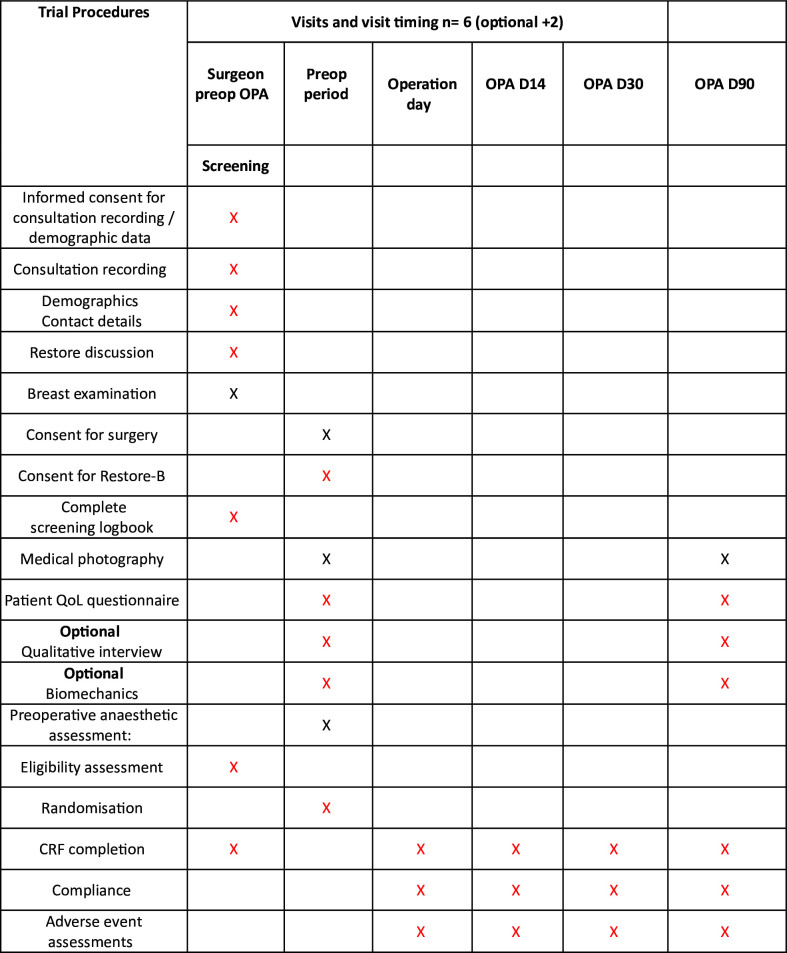
Schedule of procedures. CRF, case report form; D, day; OPA, outpatient appointment; Preop, preoperative.

Patients will be contacted at 90 days to complete an online (or paper) QoL post-operative questionnaire (in rare cases by telephone). Following completion of the 90-day QoL questionnaire, the patient will be unblinded to their allocation. Standard postoperative oncoplastic photographs will be collected at 90 days to assess breast aesthetics. Patients can report complications or additional healthcare visits to the research team via their weekly online diary. Serious adverse events will be recorded and reported if unexpected and related. Feasibility outcome data will be collected alongside clinical outcome data. Data will be collected from participants via questionnaires and CRFs. Paper questionnaires (where used) will be returned to the central trial office in Oxford via post using a preaddressed freepost envelope. Electronic questionnaires will feed directly into an online secure database—the study’s dedicated instance of REDCap (figure 2 shows the schedule of events).

####  Optional

1. Patients, when offered participation in the trial, will be in parallel offered to participate in the substudy looking to identify patient views on mesh in breast reconstruction, the design of the trial and barriers to participation in research. A patient information sheet and consent form for the qualitative interview (previously approved ethics reference: Narratives of health experience insights for https://hexi.ox.ac.uk and www.youthhealthtalk.org. b NRES Committee South Central - Berkshire 122/SC/0495. IRAS ID 112111). This is in the form of a 1-hour virtual (or face-to-face) qualitative interview with the trial research team. Patients can participate in this study regardless of their decision to take part in the main study. Participants in this substudy will be compensated for their time with a gift voucher. Deidentified transcripts will be analysed using thematic analysis, guided by a modified grounded iterative theory approach, allowing early interviews to enrich data collection in later interviews. Patients will be interviewed until analytic categories are richly covered with no further emergent themes. Three levels of coding will be used to develop conceptual analysis.
2. Patients who decide to consent to participate in the main trial will be offered the opportunity to take part in the substudy determining the feasibility of collecting breast biomechanic data presurgery and post breast reconstruction surgery. Patients will be approached preoperatively in the outpatient setting by the consenting surgeon. Trial information and trial consent for biomechanics will be supplied to patients at this visit. Participants who wish to participate will be invited to attend a preoperative and postoperative visit (at 90 days) to the biomechanics laboratory for data collection in addition to their standard NHS care visits to their hospital. Patients will need to be able to travel to the biomechanics data collection site at the University of Portsmouth. Patients will be compensated for their travel costs to the biomechanics laboratory. Participants who do consent to biomechanical data collection will have an appointment made at the University of Portsmouth breast biomechanics centre. Electronic sticker sensors (Liberty MicroSensor, Polhemus, Vermont, USA; CE Marked) will be placed onto the patient’s chest wall and breast and the patient will be asked to stand, walk slowly and walk quickly with the sensors collecting data on breast volume, shape and position statically, plus full body and breast kinematics dynamically, as well as perceptual feedback related to their breasts. Breast volume, shape and position will also be assessed statically using 3D scanning (Artec Eva 3D, Luxembourg; CE Marked). Three-dimensional motion of the body and breast will be tracked using electromagnetic sensors and data derived to calculate breast motion relative to the torso (six df). All biometric data collected is anonymous and linked to the participant study ID. Biomechanical data will be analysed by the research team. Participants in this substudy will be offered an optional questionnaire to record their views on this aspect of the trial. All participants will be given a complimentary bespoke postoperative bra according to their breast biomechanical data.

### Harms

The safety reporting window for the trial will be from the time of consent and will end at 90 days postoperatively at the end of the trial. Following the last feasibility assessment at 90 days, patients will be followed up clinically by their healthcare team as part of routine clinical NHS (National Health Service) care. Investigators’ follow-up of adverse events will be until event resolution or stabilisation. An adverse event is defined as any untoward medical occurrence in a participant to whom the intervention has been administered, including occurrences which are not necessarily caused by or related to that intervention. A serious adverse event (SAE) is defined as any untoward medical occurrence that: results in death; is life-threatening; requires inpatient hospitalisation or prolongation of existing hospitalisation; results in persistent or significant disability/incapacity; is otherwise considered medically significant by the investigator. The patient will be unblinded if they suffer a severe unexpected adverse event and knowledge of allocation is required. The PI will be able to access the operation note for the patient on their trial site electronic records and assess if a mesh was used or not. If it is not clear on the operation note, the trial statistician will access the randomisation schedule to code-break for the specific participant. The CI will remain blinded to allocation.

Only events considered related to the intervention and considered serious are recorded as an SAE. This includes any assessment results outside of normal parameters.

#### Reporting of SAEs

All side effects collected as outcomes in the CRF that also meet the definition of serious will need to be reported as an SAE during the trial’s safety reporting window. The safety reporting window is from the day of surgery (intervention) until the 90 day postoperative outpatient review at the end of the trial. Reporting must be within 24 hours of the site being aware of the SAE. Once a SAE is entered in the RedCap trial database, this automatically triggers a notification to the clinical trial unit.

##### Follow-up of SAEs

A follow-up report must be completed when the SAE resolves, when it is unlikely to change or when additional information becomes available. Follow-up information must also be provided as requested by the trial office.

### Monitoring and data safety monitoring board

Regular central monitoring will be performed by the clinical trials unit, according to the trial specific monitoring plan. Following written standard operating procedures, the monitors will verify that the clinical trial is conducted and data are generated, documented and reported in compliance with the protocol, good clinical practice (GCP) and the applicable regulatory requirements. Monthly trial management group meetings will be held to review the conduct and progress of the trial, assess safety and efficacy of the trial interventions and monitor safety of the participants. A trial steering committee will act as an independent body responsible for the overall supervision of the study on behalf of the trial sponsor. The trial steering committee will assess patient safety, conduct of the trial according to principles of GCP and the UK Clinical Trial Regulations. Meetings will take place at 6-month intervals.

### Participant timeline

Please refer to figure 2 for the trial schedule of procedures.

## Statistical considerations

### Sample size

The aim of this trial is to assess the feasibility of an RCT in no-mesh implant-based pre-pectoral breast reconstructive surgery. Therefore, to achieve this aim, the sample size is based on estimating the feasibility outcomes with the desired precision and not hypothesis testing.

Results will be reported in line with the CONSORT guidance on reporting randomised feasibility trials. No formal statistical comparison between groups is planned given the nature of the trial. The main focus of the statistical analysis is on quantifying uncertainty related to the feasibility measures: proportion eligible, consenting, randomised with data and follow-up data available. The overall implant loss, implant infection and seroma rate will be calculated to add related sample size calculations for a definitive trial. 95% CI for feasibility measure will be calculated using appropriate methods (eg, Wilson’s for binary). All outcomes will be summarised using descriptive summary statistics overall and, when collected for both arms, by treatment groups. Binary and categorical data will be summarised by frequencies and percentages. Continuous data will be summarised by means and SDs or median and IQR range if data are skewed. Visual representation of outcomes will be considered and, where it will support interpretation, presented.

The number of eligible participants in total, the number of participants that consented for inclusion, and the number of eligible participants randomised will be reported descriptively. Primary feasibility outcomes will be assessed with follow-up rates at each time point also reported.

### Recruitment

A proposed recruitment target of 40 patients across four centres is estimated according to reconstruction rates in each centre, previous surgical trial experience on recruitment rates (20%) and to allow a 10% withdrawal rate. The actual recruitment rate is unknown and part of the feasibility outcome data to be collected in this trial. Each centre will recruit for a total of 12 months to enable an estimation of actual recruitment rate for this trial. Recruitment data at 6 months will be assessed to determine if further sites are required to facilitate recruitment numbers. Recruitment commenced on the 19 March 2024 and will cease on the 31 August 2025.

### Randomisation

Randomisation will take place once informed consent has been given, eligibility has been confirmed and baseline questionnaires have been completed. The randomisation schedule will be designed by the chief trial statistician who will hold the allocation code. Randomisation will use a 1:1 ratio, via a secure web-based system developed and maintained by the Oxford Surgical Intervention Trials Unit in accordance with standard operating procedures. Stratification by centre will help to ensure that any centre effect will be equally distributed in the trial arms and enable practical issues associated with the active intervention to be overcome. This will be the only stratification factor. In the case of bilateral operations, the participant will be randomised, not each breast. The web-based system is accessed by the surgeon via RedCap. Randomisation allocation will be revealed to the surgeon online, and this will be accessed while the patient is in the anaesthetic room preparing for surgery. Selection bias at the point of recruitment will be minimised using strict inclusion criteria.

### Blinding

Due to the nature of the intervention, it is not possible to blind the surgeons for the purpose of the trial. Participants will be blinded to their allocation. On withdrawal or at the end of the trial, the patient will be informed of their allocation at 90 days postoperatively. Unblinding will occur after completion of the 90 day postoperative QoL questionnaire to reduce bias in its completion. Data will be collected on the administrative processes of randomisation, allocation concealment and the effectiveness of the blinding strategy of the patient to the allocation. Assessment of cosmesis between the two arms will be via a blinded panel of surgeons and patient public representatives using an established cosmetic scoring system.^11^ All questionnaires used in the trial (EQ5D and BREAST-Q) are validated questionnaires. Trial data analysis will be blinded to allocation to reduce bias in data interpretation.

### Data management

Participant data will be handled according to the central clinical trial unit’s standardised procedures, within the University of Oxford and will be compliant with all GDPR (General Data Protection Regulation) requirements.

### Statistical methods

#### Primary analysis

One key objective is to ascertain the recruitment rate of a definitive trial. Based on the four sites’ current reconstructive rates over a 12-month recruitment period, it is expected that there will be 15 eligible patients per month or 180 in total. Observing 180 patients allows the recruitment rate to estimate with a 95% CI width (Wilson’s method) of 9–15% depending on the event rate. Allowing for a recruitment rate of 25% of eligible patients, three to four per month, over 12 months, our sample size will be 40 patients to be recruited (20 per arm). The eligibility and recruitment rates observed in the feasibility trial will determine how many centres are required for feasible recruitment into the main trial within a reasonable timeframe.

The optimum primary endpoint for the main trial will be determined by both the variability and the completeness seen in this feasibility trial and will be decided through patient and professional qualitative interviewing and stakeholder surveys. Important long-term clinical outcomes will also be assessed within the main trial, including implant migration, mesh erosion, capsular contracture and surgery revision rates at 12 months post operation from patient records. The subsequent sample size calculation, along with the eligibility and recruitment rates observed in the feasibility trial, will guide how many centres are required for feasible recruitment into the main trial within a reasonable timeframe.

#### Secondary analyses

1. Qualitative interviewing: participant qualitative interviews as deidentified transcripts will be analysed using thematic analysis guided by a modified grounded iterative theory approach, allowing early interviews to enrich data collection in later interviews. Participants will be interviewed until analytic categories are richly covered with no further emergent themes. Three levels of coding will be used to develop conceptual analysis to identify barriers to participation in research and patient understanding of mesh in breast reconstruction. The data will be analysed using both a systematic and mind-mapping approach.^12^
2. Breast biomechanics: data from the breast biomechanics substudy are anonymous and analysed to determine differences between breast three-dimensional motion preoperatively and postoperatively between the two interventions.
3. Economic analysis: data from patient diaries, patient ranking of outcomes and results from the QoL questionnaires will inform the development of a decision model structure to represent the natural history of patients in IBBR. An early evaluation of the model using data from the feasibility study will help to inform cost-effectiveness analysis between the two trial interventions.

## Data and trial monitoring committee

As this is a feasibility trial, there is not a formal data monitoring committee; however, the trial management committee will review any concerns with data within their meetings biannually.

## Ethics and dissemination

### Research ethics approval

This trial will be conducted in accordance with the principles of the Declaration of Helsinki and in accordance with the relevant regulations and GCP. Following sponsor approval, the protocol, informed consent form and participant information sheet were submitted to an appropriate research ethics committee (REC), HRA (Health Research Authority)and the host institutions for written approval. Ethical approval was obtained from the UK Hampshire B REC committee, UK (Ref: 23/SC/0302; IRAS: 301423).

### Dissemination policy

Results of this trial will be published in a peer-reviewed medical journal. We will disseminate findings via professional organisations, for example, Association of Breast Surgeons and British Association of Plastic Surgeons. Social media, local media and feedback from our patient public involvement (PPI) advisory group will be used to maximise circulation of findings to patients and public. Co-presentation and publications will be produced with our PPI group.

### Protocol amendments

Any protocol amendments will be communicated to the relevant parties for approval (minor) TSC, sponsor and REC committee (major).

### Consent and confidentiality

Informed written consent will be obtained by operating surgeons from participants prior to their enrolment in the trial (participant consent form supplementary material). A patient information sheet will be provided on the trial prior to consent, with adequate time for the patient to consider participation prior to consent. Participant confidentiality will be upheld within the trial according to CTU (Clinical Trials Unit) and sponsor protocols.

### Post-trial care

Participant post-trial care will be according to standard NHS standard of care following breast reconstruction according to hospital site protocols.

### Trial registration

Trial registration numbers: NCT06112977; ISRCTN17470747; protocol version: 2.0.

## Discussion

There remains uncertainty as to the role of mesh in prepectoral breast reconstruction. Systematic reviews of available data for mesh in submuscular breast reconstruction have raised concerns regarding an increased risk of complications in the mesh group; mesh groups had a higher likelihood of infection (OR, 2.7; 95% CI, 1.1 to 6.4) and loss of implant (OR, 3.0; 95% CI, 1.3 to 6.8) than without mesh.^13 14^ These analyses, however, were limited due to heterogeneity between studies. Most data on effectiveness and safety of mesh are reported in small, single centre studies with substantial methodological limitations. One RCT comparing submuscular no-mesh with prepectoral mesh reported an increased rate of implant loss with mesh (OR 8·80, 95% CI 8·24 to 9·40),^15^ another reported increased overall complications with mesh, but found no difference in implant loss nor QoL between the groups.^16^ Surgeons can offer prepectoral implant-based reconstruction without mesh as an alternative technique to prepectoral IBBR with mesh.^15^ Published evidence demonstrates acceptable patient outcomes for this technique and is gaining popularity amongst reconstructive surgeons aiming to reduce mesh-related complications and direct costs for the operation.^17^ It is uncertain how removing the mesh impacts on patient outcomes including QoL, as data are limited to observational case series.

### Evidence gaps exist in breast reconstruction.

A systematic review concluded that there is a major unmet need for a high-quality RCT to address the uncertainty regarding the effectiveness and safety of mesh-based reconstruction.^18^ There have been no published RCTs looking at mesh vs no-mesh in prepectoral breast reconstruction. The Restore-B trial aligns with multistakeholder priorities (Association of Breast Surgeons UK and Independent Cancer Patients’ Voice UK), with a consensus identifying research generating evidence on clinical and cost-effectiveness in breast reconstruction as a key research priority.^19^ As there is a paucity of high-quality data to inform clinicians and patients regarding the use of mesh in this operation, past surgical experience and training form the main driving factor for decision making. This results in heterogeneous practice and a lack of centralised monitoring of surgical and patient-reported outcomes to adequately compare the two techniques.

This feasibility trial aims to assess if it is possible to conduct a future RCT comparing no mesh to mesh-assisted IBBR. It will examine patient and clinician equipoise towards the two techniques and establish which outcomes matter most to patients and surgeons. The feasibility data will inform the decision to proceed to the larger RCT. Results from a future RCT in this field would enable informed evidence-based treatment decisions on the use of mesh and may identify subgroups of patients who derive differential benefit from mesh: for example, those with larger breast volumes who may require additional support. This evidence will allow the approach to breast reconstruction surgery to be personalised and avoid a ‘one size fits all’ approach. Currently, it is not clear if patients and surgeons would be willing to take part in this type of trial and therefore this Restore-B feasibility trial is required. The Restore-B feasibility trial aims to address key uncertainties, which in turn may influence the success of an application for a major RCT, which would require substantial investment. It will explore areas for optimisation in trial processes, including recruitment, digital technology in trials and novel breast biomechanics.

## Data Availability

Data are available upon reasonable request.
