# Supplementary material for: Prepectoral no mesh versus mesh immediate implant-based reconstruction after mastectomy (Restore-B): a multicentre single-blinded randomised controlled feasibility study protocol
Source: BMJ Open. 2025 Oct 17;15(10):e103278. doi: 10.1136/bmjopen-2025-103278 (PMC12542532; doi:10.1136/bmjopen-2025-103278)
Supplement: Supplementary file 1 [file bmjopen-2025-103278.reviewer_comments.pdf]

PEER REVIEW HISTORY

BMJ Open publishes all reviews undertaken for accepted manuscripts. Reviewers are asked to complete a checklist review form and are provided with free text boxes to elaborate on their assessment. These free text comments are reproduced below.

ARTICLE DETAILS

Title (Provisional)

Prepectoral no mesh versus mesh immediate implant-based reconstruction after mastectomy (Restore-B): a multicentre single blinded randomised controlled feasibility study protocol.

Authors

Rolph, Rachel; Ziebland, Sue; Cook, Jonathan Alistair; Iglesias, C; Wakefield-Scurr, Joanna; Scaife, Jessica; Taylor, Amy; Malyon, Charles; Hennessy, Aimee; Markham, Sarah; Bernstein, Marcelle; Douek, Michael; Collaborative Group, Restore B

VERSION 1 - REVIEW

**Reviewer**	**1**
**Name**	**Zeeshan, Sana**
**Affiliation**	**The Aga Khan University, Department of Surgery**
**Date**	**06-May-2025**
**COI**	**None**

Well-designed study.

**Reviewer**	**2**
**Name**	**Heil, Joerg**
**Affiliation**	**University Hospital Heidelberg**
**Date**	**25-May-2025**
**COI**	**None**

Excellent paper on a feasability study on a very, very important subject in breast reconstruction. Perfect trial strategy.

VERSION 1 - AUTHOR RESPONSE

Reviewers:

-
  Reviewer: 1
  Dr. Sana Zeeshan, The Aga Khan University
  Comments to the Author: Well-designed study.
- Reviewer: 2
  Dr. Joerg Heil, University Hospital Heidelberg
  Comments to the Author:
  Excellent paper on a feasability study on a very, very important subject in breast reconstruction. Perfect trial strategy.

We thank the reviewers for their positive and supportive comments on our protocol. No changes required.
